# PEDOT Composite with Ionic Liquid and Its Application to Deformable Electrochemical Transistors

**DOI:** 10.3390/gels8090534

**Published:** 2022-08-25

**Authors:** Sangkyu Lee, Jaepyo Jang, Sungjun Lee, Daekwang Jung, Mikyung Shin, Donghee Son

**Affiliations:** 1 Department of Electrical and Computer Engineering, Sungkyunkwan University (SKKU), Suwon 16419, Korea; 2 Center for Neuroscience Imaging Research, Institute for Basic Science (IBS), Suwon 16419, Korea; 3 Center for Bionics of Biomedical Research Institute, Korea Institute of Science and Technology, Seoul 02792, Korea; 4 School of Electrical and Electronic Engineering, Yonsei University, Seoul 03722, Korea; 5 Department of Intelligent Precision Healthcare Convergence, Sungkyunkwan University (SKKU), Suwon 16419, Korea; 6 Department of Biomedical Engineering, Sungkyunkwan University (SKKU), Suwon 16419, Korea; 7 Department of Superintelligence Engineering, Sungkyunkwan University (SKKU), Suwon 16419, Korea

**Keywords:** PEDOT:PSS, ionic liquid, organic electrochemical transistor, stretchable electronics, skin-inspired electronics

## Abstract

Organic electrochemical transistors (OECTs) have become popular due to their advantages of a lower operating voltage and higher transconductance compared with conventional silicon transistors. However, current OECT platform-based skin-inspired electronics applications are limited due to the lack of stretchability in poly(3,4-ethylenedioxythiophene):polystyrene sulfonate (PEDOT:PSS). Some meaningful structural design strategies to resolve this limitation, including rendering OECT to make it more stretchable, have been reported. However, these strategies require complicated fabrication processes and face challenges due to the low areal density of active devices because wavy interconnect parts account for a large area. Nevertheless, there have been only a few reports of fully deformable OECT having skin-like mechanical properties and deformability. In this study, we fabricated stretchable and conductivity-enhanced channel materials using a spray-coating method after a composite solution preparation by blending PEDOT:PSS with several ionic liquids. Among these, the PEDOT composite prepared using 1-butyl-3-methylimidazolium octyl sulfate exhibited a better maximum transconductance value (~0.3 mS) than the other ion composites. When this material was used for our deformable OECT platform using stretchable Au nanomembrane electrodes on an elastomer substrate and an encapsulation layer, our d-ECT showed a barely degraded resistance value between the source and drain during 1000 cycles of a 30% repeated strain. We expect that our d-ECT device will serve as a step toward the development of more precise and accurate biomedical healthcare monitoring systems.

## 1. Introduction

Organic electrochemical transistors (OECTs) have received considerable attention for their wearable skin-inspired electronics and implantable bioelectronics [1,2,3,4,5,6,7]. They can operate in wet environments such as biofluids or cells because of their ionic conductive electrolyte layer and soft channel polymers. Although this operation principle is different from conventional silicon-based transistors using rigid inorganic dielectric and semiconductors, OECTs show a lower operation voltage and higher transconductance characteristics attributed to the high volumetric capacitance at the interface between the electrolyte and channel polymers in contrast with traditional organic thin-film transistors [8,9]. Based on these characteristics, OECTs are preferred as biosensing tools as they can be integrated with skin, nerves, and various other tissues and operate as highly sensitive active sensor devices that are capable of detecting the micro-oscillations in electrophysiological signals and amplifying an input into a large output [9,10,11,12,13,14,15,16]. However, most efforts to study OECT devices have focused on the synthesis of novel, highly conductive, and electrochemically stable channel materials [17,18,19] or the physical modeling of devices [20,21,22]. However, the research on soft and deformable OECTs has progressed recently [22,23,24,25,26].

This critical problem results from the limited features of poly (3,4-ethylenedioxythiophene):polystyrene sulfonate (PEDOT:PSS), which is the most widely used material in OECT channels. PEDOT:PSS doped with anionic PSS in the semiconducting polymer PEDOT has both electrical and ionic conductivity, facilitating low electrochemical impedance properties under various solid or liquid electrolyte environments. Owing to this bio-friendly charge transport property, PEDOT:PSS is widely used as a conducting material for electrode devices and a channel material in various OECT devices for biosensors and electrophysiological sensors [27,28,29]. However, PEDOT:PSS cannot easily achieve conformal and adaptive integration with bio-tissues due to its intrinsically low deformability [30,31]. To overcome this material constraint, certain efforts have been dedicated to functionalizing PEDOT:PSS; these efforts have involved combining chemically modified groups or blending physical polymer matrices to impart stretchability while maintaining the established electrochemical properties of the original PEDOT:PSS [32,33,34,35]. As a representative finding, PEDOT composites with softness, stretchability, and enhanced conductivity functionalized by incorporating ionic additive-assisted stretchability and electrical conductivity (STEC) enhancers were developed by blending sulfonic or imidazole-based ionic liquids with PEDOT:PSS [35]. Nevertheless, there are still a few reports about the skin-like soft and deformable OECT based on these stretchable PEDOT composite channels.

**Figure 1:**
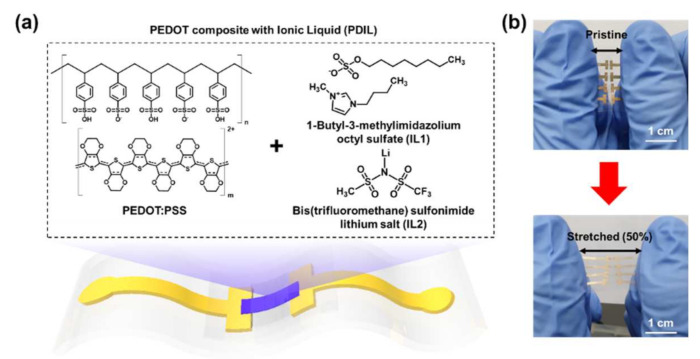
Schematic of our deformable electrochemical transistor (d-ECT) fabricated using a PEDOT composite with an ionic liquid channel. (**a**) Conceptual design of our d-ECT based on PEDOT:PSS and several ionic liquids. (**b**) Photograph of the d-ECT device.

In this study, we developed a stretchable PEDOT composite film with ionic liquids (PDIL) through spray coating for a deformable organic electrochemical transistor (Figure 1). We optimized the PDIL channel through ionic liquid selection and fabricated a deformable electrochemical transistor (d-ECT) device employing a PDIL channel and microcrack-based stretchable Au electrode without a wavy interconnect pattern. In the channel optimization process, the PEDOT composite with a 1-butyl-3-methylimidazolium octyl sulfate-based PEDOT:PSS channel exhibited the maximum transconductance (~0.3 mS) among several channel materials when the ECT was fabricated on a glass plate. After the selection of the optimum channel, our d-ECT showed its electrical stability over 1000 cycles at a 30% strain. Thus, we believe that the development of our d-ECT can contribute to the establishment of key fundamental technologies for next-generation skin-inspired electronics [36,37,38,39,40,41,42] for biomedical applications and healthcare platforms [43,44,45,46,47,48,49,50] and are capable of adaptive conformal integration with human skin [51,52,53].

## 2. Results and Discussion

### 2.1. Optimization of the Spray-Coating Volume of PEDOT:PSS on an Elastomer Substrate

**Figure 2:**
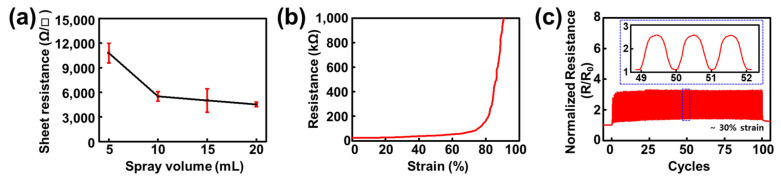
Characterization of poly(3,4-ethylenedioxythiophene):polystyrene sulfonate (PEDOT:PSS) spray-coated onto a poly(styrene-ethylene-butylene-styrene (SEBS) substrate. (**a**) PEDOT:PSS spray-volume optimization through sheet resistance saturation. (**b**) Continuous stretching test of 10 mL PEDOT:PSS on SEBS substrate. (**c**) Cyclic stretching test of 10 mL PEDOT:PSS on SEBS.

Prior to investigating the stretchable channel for the d-ECT device, we hypothesized that the electrical properties of the spray-coated bare PEDOT:PSS would be saturated at a specific volume. However, we were uncertain whether our elastomer, poly(styrene-ethylene-butylene-styrene) (SEBS) could endure high-temperature conditions (170 °C) during the spray-coating process. Therefore, we first sprayed a bare PEDOT:PSS solution with methanol (1:4 *v*/*v*) on a glass substrate. After confirmation of the temperature stability of SEBS under these conditions, we optimized the spray volume of the PEDOT:PSS on the glass plate from 5 to 20 mL to identify the minimum spray volume condition of PEDOT:PSS that could cause saturated electrical performance. Consequently, the sheet resistance of the sprayed PEDOT:PSS film was found to saturate at a spray volume of 10 mL (Figure 2a). After this optimization, all the PDIL composites were sprayed at 10 mL in all the measurement procedures.

Next, we confirmed the electrical stability of the PEDOT:PSS films spray-coated onto SEBS under several stretching conditions. During continuous stretching, the electrical resistance change was found to be negligible at a ~70% strain; however, it reached electrical breakdown at an 80% strain (Figure 2b). Thus, the films exhibited superior stretching performance to the previously reported bare PEDOT:PSS films (~10% fracture strain) [31]. Notably, human skin can generally be reversibly stretched to 15% and irreversibly stretched to a maximum of approximately 30% [54]. Therefore, we assumed that spray-coated PEDOT:PSS would show stable electrical performance during cyclic stretching. To validate this assumption, we conducted a cyclic stretching test on PEDOT:PSS sprayed onto SEBS at the stretching scale of the skin (Figure 2c). As expected, the resistance change in the PEDOT:PSS sprayed on SEBS was continuously maintained between 20 and 50 kΩ over 100 cycles. The minimum resistance slightly increased from 15 kΩ in the initial state to 20 kΩ in the first cycle; however, this value was nearly constant during the final 100 cycles, with a final resistance of 20.7 kΩ.

From these results, we hypothesized that the PDIL composites, which are stretchable PDIL ion composites reported previously [35], could display a better electrical or mechanical performance as a d-ECT channel than bare PEDOT:PSS. The developed d-ECT with PDIL follows the flow chart (Figure S1).

### 2.2. Comparison of PDIL Composites for the Selection of Channel Materials to Be Used in ECT

To identify the optimal channel material among the stretchable PEDOT derivatives, we first selected two ionic liquids, 1-butyl-3-methylimidazolium octyl sulfate (IL1) and bis(trifluoromethane) sulfonimide lithium salt (IL2) because these ionic liquid-based PEDOT films demonstrate better conductivity or maximum tensile strain values than the other ionic liquids presented in the freestanding bulk film state comparison table obtained from a previous report [35]. Subsequently, we sprayed each PDIL solution and the bare PEDOT:PSS solution as a control on the Au source/drain (S/D) electrodes placed on a rigid glass plate. To deposit the channel region between the source and drain, we attached a custom-made shadow mask to the glass plate using adhesive polyimide tape before spray coating (Figure S2).

**Figure 3:**
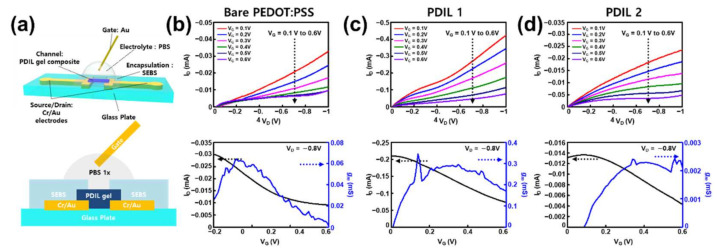
Device characterizations of ECT using bare PEDOT:PSS, PDIL 1, and PDIL 2 channels on a glass substrate (Channel Length: 0.5 mm, Width: 1.2 mm, Thickness: 1 μm). (**a**) Measurement setup of rigid electrochemical transistors for channel investigation. (**b**) Output and transfer characteristics of the ECT device using the bare PEDOT:PSS channel. (**c**) Output and transfer characteristics of the ECT device using the PDIL 1 channel. (**d**) Characterizations of ECT using bare PEDOT:PSS, PDIL 1, and PDIL 2 channels on a glass substrate (Output and transfer characteristics of the ECT device using the PDIL 2 channel).

After the deposition of the PDIL composites, an additional SEBS encapsulation layer was transferred, and the channel exposure region was plasma-etched with oxygen to protect the Au S/D electrodes from the electrolyte and expose the channel region to establish contact with the electrolyte. The encapsulation layer was essential to prevent the gate-drain short (Figure S3). In other words, the Au-electrolyte charge transport was considered to be more dominant than the electrochemical reaction at the PEDOT:PSS–electrolyte interface because the resistance of the Au pads and interconnects was smaller than that of PEDOT:PSS. After encapsulation, a phosphate-buffered saline solution (PBS) was dropped as a liquid electrolyte into the etched hole on the encapsulation layer (Figure 3a) for the preparation of the electrochemical transistor device (ECT).

All of our devices (ECTs and d-ECTs) had the same working principle: A low positive gate voltage was applied, and anions on the electrolytes moved into the gate–electrode surface. An electrical double layer (EDL) at the gate–electrolyte interface was formed by these anions to neutralize (or compensate) the positive charges. On the contrary, cations on the electrolytes migrated to the semiconducting channel polymer. When this migration occurred, the swelling of the PSS chain of the PEDOT:PSS was caused by aqueous PBS electrolytes so that cations could penetrate and diffuse into the PEDOT:PSS channel. In this process, another EDL at the electrolyte–semiconductor interface was formed by migrated cations in the same manner. Due to these EDLs, a volumetric capacitance, a unique characteristic of OECTs, was displayed and contributed to a lower voltage operation or high transconductance. However, at the same time, additional electrochemical redox reactions occurred. PEDOT:PSS is an intrinsically highly-doped (oxidized) semiconductor, but it can be dedoped (or oxidized) by interdiffused cations and neutralized (channel conductivity decreases).

$$PEDOT^{+}:PSS^{-}+\left[Cat^{+}\right]\to PEDOT^{0}+\left[Cat^{+}\right]:PSS^{-}$$

If the negative gate voltage was applied, the opposite process, the doping process (or reduction), reversibly occurred. This electrochemical redox reaction makes the OECTs significantly different from the electrolyte-gated field-effect transistors that also use liquid electrolytes [2,3].

Following the preparation of these ECTs, their transfer characteristics and output characteristics were measured for performance characterization on a glass plate (Figure 3b–d and Figure S4). These characteristics were measured to evaluate the performance of the transistor devices. In general, the transfer characteristics are correlated to on/off switching (drain current dependency on the gate voltage) and the output characteristics are related to the amplifying function in the ON state of transistors (drain current dependency on a given drain voltage). Unlike conventional field-effect transistors, transconductance is more emphasized as a figure-of-merit of OECT devices than electrical mobility. A transconductance (*g_m_*) generally means the conversion efficiency of the applied gate voltage into the drain current change that is calculated by

$$g_{m}=\frac{\partial I_{D}}{\partial V_{G}}\;(1\mathrm{st}\;\mathrm{derivative}\;\mathrm{of}\;\mathrm{the}\;\mathrm{transfer}\;\mathrm{curve})$$

In other words, *g_m_* expresses a variance in the slope of the transfer curve. The steeper the transfer curve, the larger the drain current change for an applied gate voltage. When the OECT is used as a biosensor, this *g_m_* means a factor that is related to a sensitivity to target analytes [1,3,5,6].

As a result of these characterizations, PDIL 1 (PEDOT:PSS with 1-butyl-3-methylimidazolium octyl sulfate) demonstrated improved drain current and transconductance than the bare PEDOT:PSS and PDIL 2 (PEDOT:PSS with bis(trifluoromethane) sulfonimide lithium salt). Therefore, we concluded that this PDIL 1 could serve as the best channel material for the ECT device (Figure 3c). The maximum transconductance was approximately 0.3 mS, which is fivefold higher than that of bare PEDOT:PSS (Figure 3b). In contrast, PDIL 2 did not act as a channel material in the ECT device (Figure 3d). We assumed that this was due to the presence of lithium, a metallic component in the ionic liquid 2 (bis(trifluoromethane) sulfonimide lithium salt). Since the PDIL 2 channel was a metal-doped channel, the on/off ratio was the lowest (approximately ~7) among the other ECTs so its transconductance (~0.003 mS) was also much lower than the others because of its low on/off ratio. Interestingly, it was observed that the continuous stretching tests of these PDILs on SEBS also showed a similar trend and the PDIL 1 on SEBS kept its electrical resistance with a slight increase (but it was still under 1 kΩ at a 100% strain) so it showed better stretchability (>100%) than the other channels (Figure S5).

### 2.3. Electrical Performance of d-ECT during Stretching

**Figure 4:**
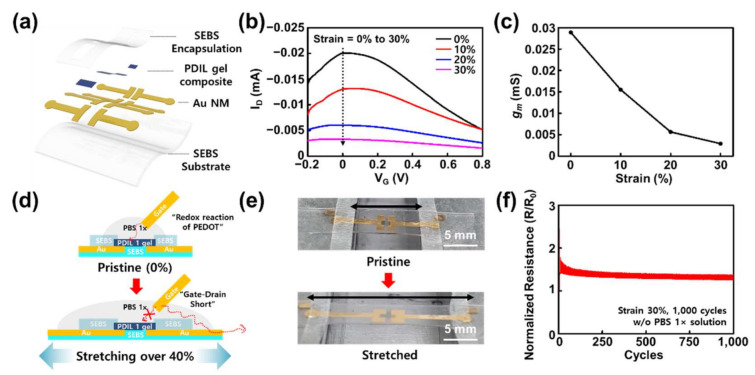
Electrical performance stability of d-ECT during the stretching test. (**a**) Each layer on the d-ECT device (channel length: 0.5 mm, width: 1.2 mm, thickness: 1 μm). (**b**) Transfer curve changes in the d-ECT owing to the applied strain. (**c**) Maximum transconductance changes in the d-ECT owing to the applied strain. (**d**) Gate–interconnect short effect caused by 40% strain. (**e**) Photograph of pristine and stretched d-ECT on a custom-built stretcher system. (**f**) Cyclic stretching test of d-ECT without liquid electrolyte during 1000 cycles of 30% strain.

After the rigid substrate test of our PDIL channels, we fabricated a deformable d-ECT using an SEBS substrate with evaporated Au source/drain electrodes. After spray coating the PDIL 1, the spin-coated SEBS layer was transferred from the ODTS-treated silicon wafer, and the channel exposure region was exposed through plasma etching using a shadow mask (Figure 4a). As mentioned, the bare PEDOT:PSS on SEBS was stretchable at a ~70% strain and exhibited only slight resistance changes (Figure 2b). Therefore, we expected the deformability of our d-ECT using the PDIL 1 composite channel to also be better than the bare PEDOT:PSS on SEBS.

When the d-ECT was stretched along the channel length direction, the transfer characteristics and maximum transconductance value sharply reduced owing to the strain (Figure 4b,c). We hypothesized that this steep decrease could be attributed to liquid electrolytes. This meant that, as a result of the applied strain, the PBS electrolyte solution droplet gradually spilled from the top of the encapsulation layer to the Au interconnects, causing an electrical short (Figure 4d and Figure S6). Indeed, there were negligible changes in their electrical resistances between the source and drain electrode when the d-ECT device without a liquid electrolyte was stretched over 1000 cyclic tests from a 0% to 30% strain and a 50% strain (Figure 4e,f and Figure S7). It consequently showed that our d-ECT had reliable electrical stability in harsh stretching conditions without liquid electrolytes. In addition, the surface morphology of the PDIL 1 composite channel region also showed that the PDIL 1 composite had smaller cracks than the other channels when they were 30% stretched (Figure S8).

## 3. Conclusions

In this study, we applied several PDIL composites as d-ECT channel materials through spray coating, which is the simplest among the various deposition methods. We first validated that our device platform (direct spray-on elastomer substrate) was valid despite the high-temperature conditions of the spray-coating process (170 °C). The sprayed PEDOT:PSS on SEBS substrate was stretchable at a ~70% strain and exhibited only slight resistance changes in a cyclic test at a 30% strain. Consequently, 1-butyl-3-methylimidazolium octyl sulfate was identified to exhibit the highest transconductance value (~0.3 mS). By spraying this optimized channel material (PDIL 1) onto stretchable metal electrodes on an elastomer substrate, we fabricated a deformable d-ECT device. Our d-ECT device exhibited electrical stability in a cyclic strain test of over 1000 cycles without a liquid electrolyte at a 30% strain and even at a 50% strain. However, the liquid electrolyte used in our d-ECT was challenging because of its contact with the interconnects under strain for the realization of a fully stretchable ECT device. Another obstacle was the electrical performance of our d-ECT device so it needs further improvements compared to the recently reported deformable or stretchable OECT devices (Table S1). After these problems are solved, we expect that our d-ECT will find a wide range of applications extending from various wearable skin devices to bioelectronic circuits for wearable and implantable healthcare monitoring systems.

## 4. Materials and Methods

### 4.1. Thermal Evaporation of Source/Drain Electrodes on a Stretchable Substrate

All solvents were purchased from Sigma-Aldrich (Burlington, VT, USA). The Au–SEBS electrodes were prepared according to a previous study [55,56,57]. Briefly, cleaned 100 mm × 100 mm glass plates (JMC glass, Ansan, Korea) were prepared as stretchable substrates for the source/drain electrodes. The SEBS (Tuftec^TM^ H1062, Asahi Kasei Co., Tokyo, Japan) solution (150 mg/mL in chloroform) was drop-cast on top of a glass substrate and dried overnight. Three stainless-steel shadow masks with patterns (for electrodes, channel, and encapsulation layers) were prepared using laser patterning equipment. A custom shadow mask (approximately 80 mm × 80 mm) with electrode patterns was attached to the SEBS film on the glass substrate using commercial Kapton tape (KT-10, Bedell Co., Seoul, Korea) to create source/drain electrodes on the glass or SEBS substrates.

Au nanomembrane-based stretchable electrodes (50 nm-thickness) were directly deposited onto the glass plate or SEBS film surface following the custom shadow mask patterns during thermal evaporation. After thermal evaporation, the polyimide tape was removed and the custom shadow mask was also removed from the SEBS film glass plate. A digital multimeter was used to check whether every source/drain electrode was electrically short or not.

### 4.2. Preparation of the PDIL Channel and SEBS Encapsulation Layer

PEDOT: PSS (Clevios PH1000, Heraeus, Germany) was used as the initial solution to prepare the PDIL composite. Each selected ionic liquid was mixed in an appropriate ratio according to a previous report [35]. In detail, we chose 1-butyl-3-methylimidazolium octyl sulfate for PDIL 1 and bis(trifluoromethane) sulfonimide lithium salt for PDIL 2. Methyl alcohol was used as a diluent for all PEDOT:PSS-based solutions (PH1000:Methyl Alcohol = 1:4 *v*/*v* ratio) to prevent jamming of each PDIL composite solution on the spray-gun nozzle. ODTS-treatment of the silicon wafer was prepared by immersion of n-hexane and an annealing process following the previous report [37].

During the spray-coating process, a commercial spray gun (HP-BCP plus airbrush, Iwata, Japan) and purified nitrogen gas (JC gas, Anseong, Gyeonggi-do, Korea) were used (spray pressure: 40 psi). An aluminum foil was used to encapsulate the hot plate to prevent contamination of the hot plate surface. A secondary custom shadow mask was attached to the sample plates to isolate the channel region between the source and drain electrodes during spray coating. The spraying distance between the sample glass plates placed on the hot plate (170 °C) and the hand-grasped spray gun was approximately 15 cm. After spray coating, a PDIL channel was deposited between the source and drain electrodes. Finally, the aluminum foil was peeled off after the hot plate was turned off. A digital multimeter was used to check whether every source/drain electrode was electrically short by the PDIL channel.

To evaluate the performance of the d-ECT device, we used the SEBS encapsulation layer via spin-coating (1000 rpm, 1 min) of SEBS solution (70 mg/mL in chloroform) and transferred it to the Au S/D electrode-deposited glass or SEBS substrates. After transfer of the encapsulation layer, oxygen plasma etching (300 watts, 90 s) was conducted using a third custom shadow mask to open the channel only and to protect the interconnects from the gate leakage current. Through encapsulation, the d-ECT devices with PDIL composite channels were finally prepared.

### 4.3. Characterization of the Electrochemical Transistors (ECTs and d-ECTs)

A parameter analyzer (Agilent 4155C, Solon, OH, USA) was used to evaluate the transfer/output characteristics of all devices. A PBS 1X solution was used as the liquid electrolyte for all measurements. Each pad of the Au S/D electrodes was electrically contacted with each probe using a liquid metal (EGaIn). A digital Keithley 2450 source meter (Tektronix, Inc., Clackamas, OR, USA) was used to characterize the electrical stability test of all samples during the various stretching tests. In the first stretching test of our d-ECT, the custom-built manual stretcher stage was covered with double-sided adhesive tape (3M commercial tape). The encapsulation tape was applied to the interconnect part, and the PBS solution was dropped after the fabricated d-ECT was placed on the stretcher on each side. Continuous stretching and cyclic stretching tests of d-ECT without the PBS solution were conducted using a motor-based one-axis stretcher.

## Data Availability

The data presented in this study are available in this article.

## Supplementary Materials

The following supporting information can be downloaded from https://www.mdpi.com/article/10.3390/gels8090534/s1, Figure S1. Flowchart of the tests and characterizations for development of d-ECT devices; Figure S2. Shadow mask preparation and spray-coating environment using custom-masked shadow mask for channel deposition; Figure S3. No encapsulation effect on ECT characteristics on a glass plate; Figure S4. Transfer characteristics of ECTs on a glass plate for comparison of each PDIL channel; Figure S5. Continuous stretching test of bare PEDOT:PSS, PDIL 1 and 2 on SEBS; Figure S6. Transfer characteristics of d-ECT using PBS solution electrolyte at stretching at 40%; Figure S7. Cyclic stretching test of d-ECT without liquid electrolyte during 1000 cycles at a 50% strain; Figure S8. Scanning electron microscopy of PDIL channels on d-ECT when they were pristine and 30% stretched; Table S1. Comparison of the figures of merit of the state-of-the-art stretchable OECTs in the present work. References [15,23,24,26,58,59,60] are cited in the Supplementary Materials.
