# Supplementary material for: PEDOT Composite with Ionic Liquid and Its Application to Deformable Electrochemical Transistors
Source: Gels. 2022 Aug 25;8(9):534. doi: 10.3390/gels8090534 (PMC9498364; doi:10.3390/gels8090534)
Supplement: Supplementary file 1 [file gels-08-00534-s001.zip › gels-1863673-supplementary.pdf]

Supplementary Materials

PEDOT Composite with Ionic Liquid and Its Application to Deformable Electrochemical Transistors

Sangkyu Lee [1,2,†], Jaepyo Jang [1,2,†], Sungjun Lee [3,4], Daekwang Jung [1,2], Mikyung Shin [2,5,6,*] and Donghee Son [1,2,7,*]

1 Department of Electrical and Computer Engineering, Sungkyunkwan University (SKKU), Suwon 16419, Korea

2 Center for Neuroscience Imaging Research, Institute for Basic Science (IBS), Suwon 16419, Korea

3 Center for Bionics of Biomedical Research Institute, Korea Institute of Science and Technology, Seoul 02792, Korea

4 School of Electrical and Electronic Engineering, Yonsei University, Seoul 03722, Korea

5 Department of Intelligent Precision Healthcare Convergence, Sungkyunkwan University (SKKU), Suwon 16419, Korea

6 Department of Biomedical Engineering, Sungkyunkwan University (SKKU), Suwon 16419, Korea

7 Department of Superintelligence Engineering, Sungkyunkwan University (SKKU), Suwon 16419, Korea

* Correspondence: mikyungshin@g.skku.edu (M.S.); daniel3600@g.skku.edu (D.S)

†These authors contributed equally to this work.

Supplementary Figures S1–S8, Table S1.

Supplementary Figure S1

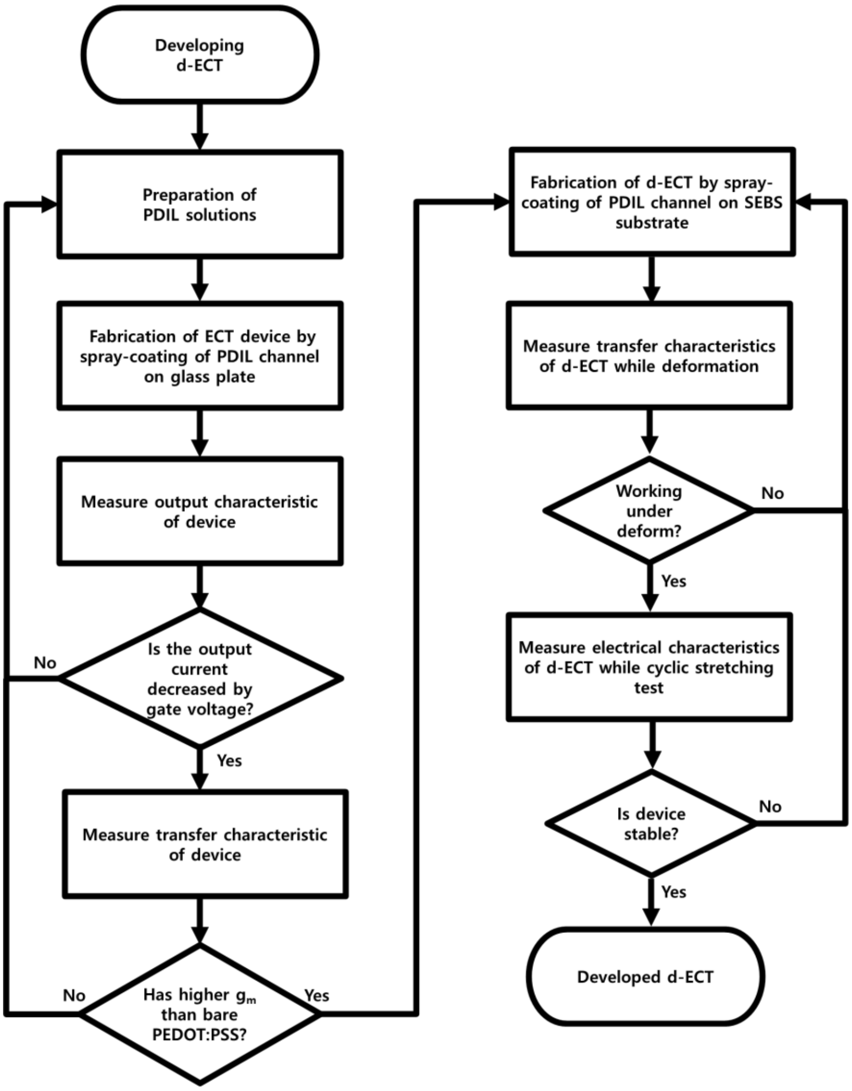

Supplementary Figure S1. Flowchart about the tests and characterizations for development of d-ECT devices.

Supplementary Figure S2

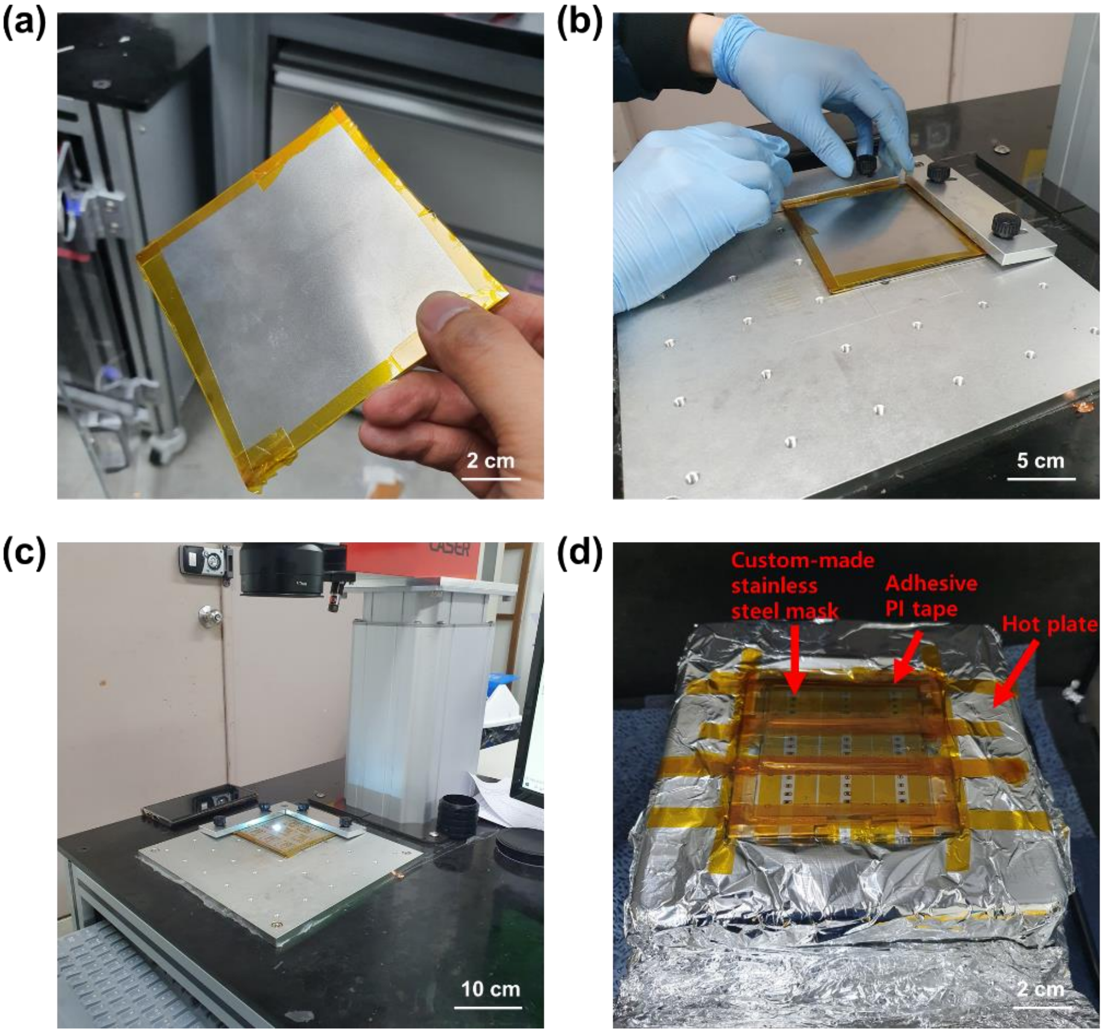

Supplementary Figure S2. Shadow mask preparation and spray-coating environment using custom-masked shadow mask for channel deposition. (a) Preparation of stain-less steel plate. (b) Fixation of plate on stage of laser-patterning equipment. (c) Laser-patterning process. (d) Spray-coating conditions.

Supplementary Figure S3

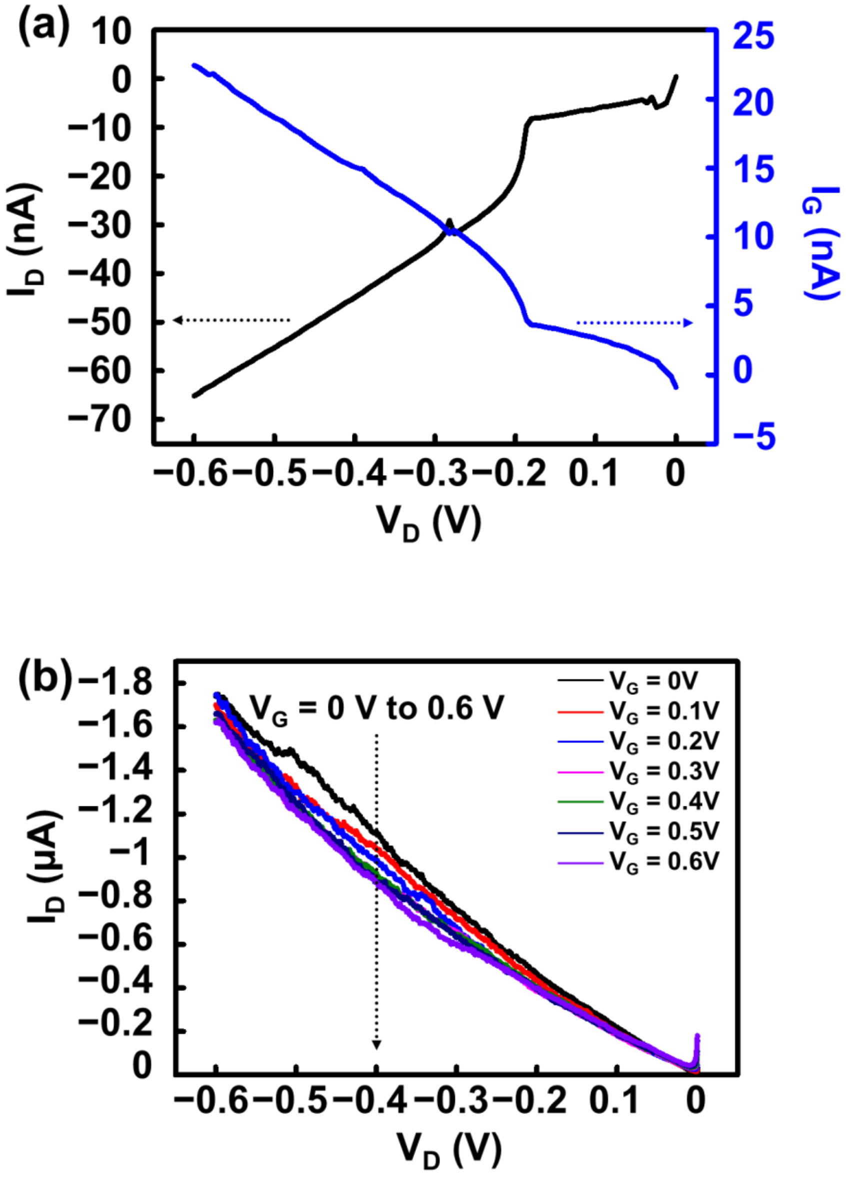

Supplementary Figure S3. No encapsulation effect on ECT characteristics on glass plate. (a) gate-drain short caused by no encapsulation of ECT. (b) transfer characteristics of ECT without encapsulation. Gate voltage could not influence the conductivity of channel (Electrochemical doping-dedoping process of PEDOT:PSS was not caused).

Supplementary Figure S4

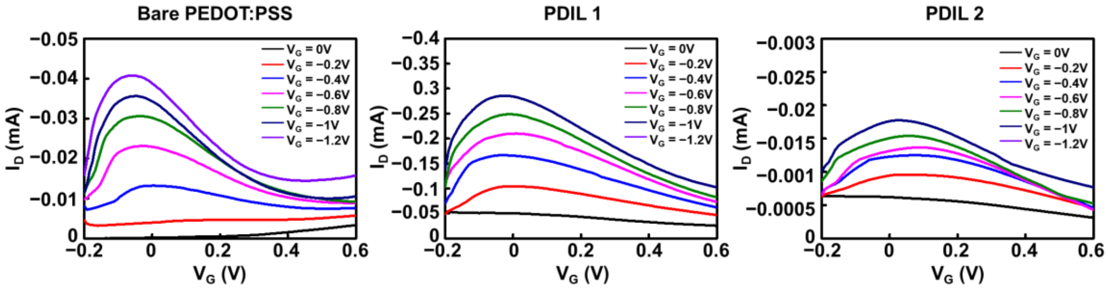

Supplementary Figure S4. Transfer characteristics of ECTs on glass plate for comparison of each PDIL channel.

Supplementary Figure S5

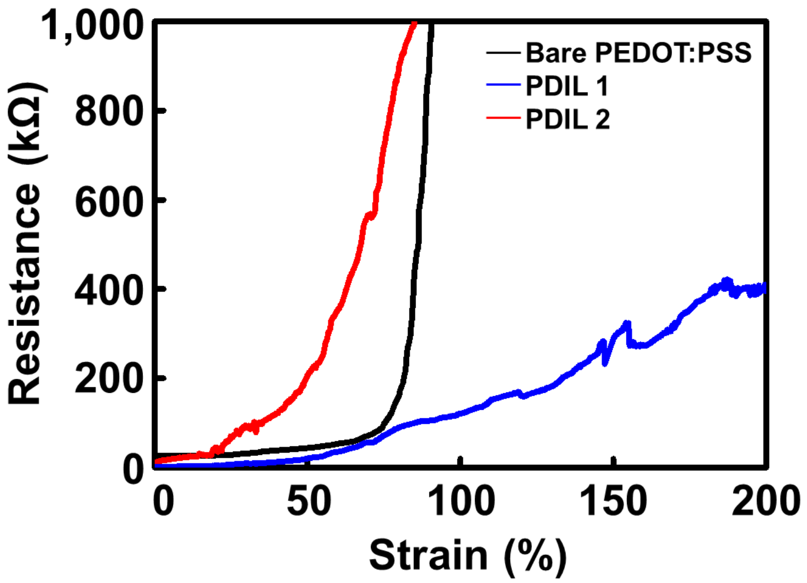

Supplementary Figure S5. Continuous stretching test of each channel material (Bare PEDOT:PSS, PDIL 1 and 2) on SEBS.

Supplementary Figure S6

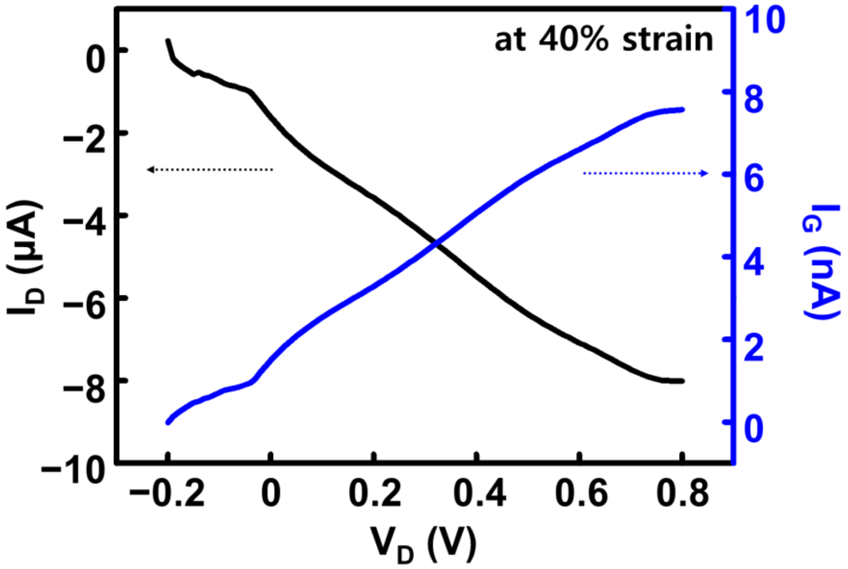

Supplementary Figure S6. Transfer characteristics of d-ECT using PBS solution electrolyte at stretching 40%.

Supplementary Figure S7

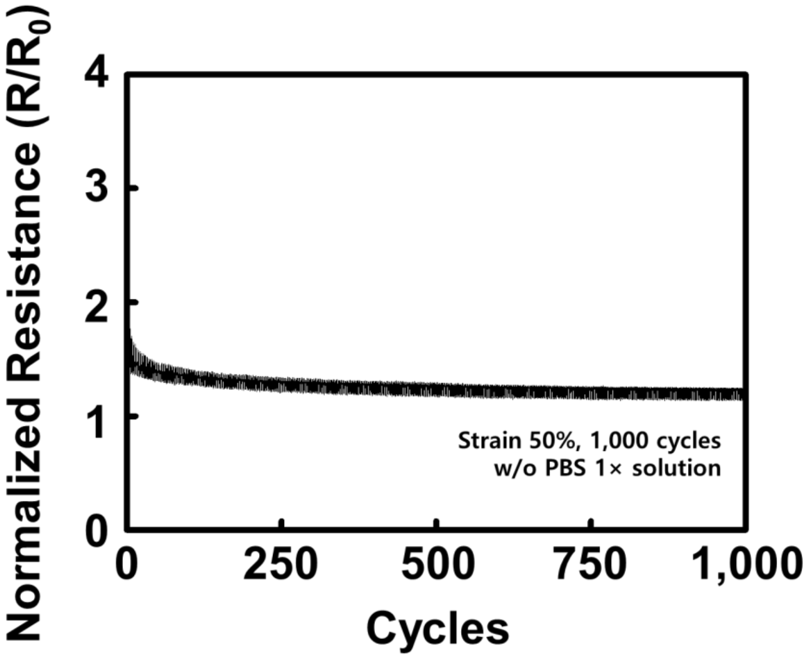

Supplementary Figure S7. Cyclic stretching test of d-ECT without liquid electrolyte during 1,000 cycles of 50% strain.

Supplementary Figure S8

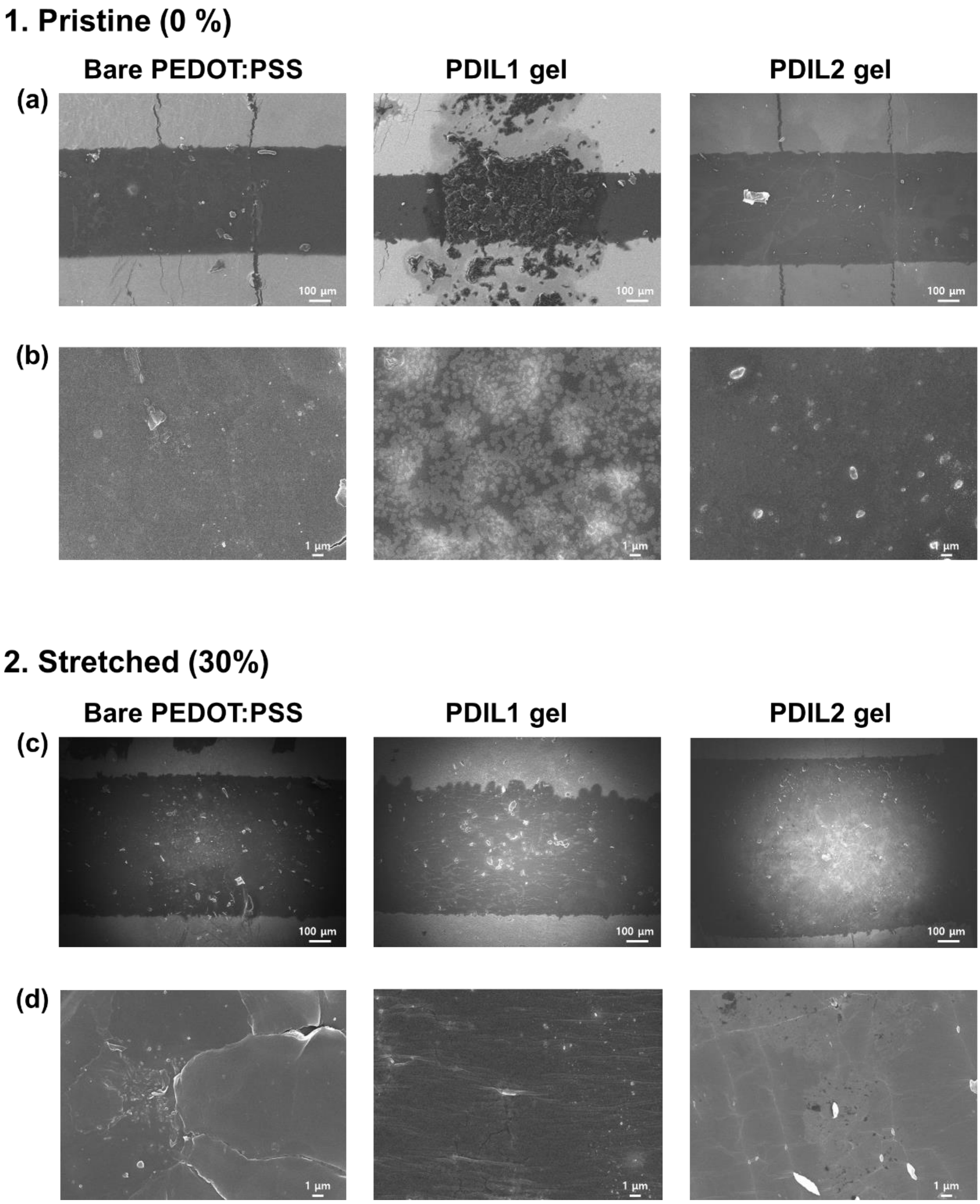

Supplementary Figure S8. Scanning electron microscopy (SEM) of PDIL channels on d-ECT when they were pristine and 30% stretched.

.

Supplementary Table S1

	Reference	Channel	Electrode	Max transconductance (mS)	* Normalized Transconductance (S/cm⁻¹)	Stretchability (%)
Extrinsic stretchable	Marchiori t al.	PEDOT : PSS	Au	6.5	NA	38
	Lee et al.	PEDOT : PSS	Au	1	NA	15
	Zhang et al.	PEDOT : PSS	Au	0.6	NA	30
Intrinsic stretchable	Dai et al.	P(g2T-t)	Vertical Au nanowires (Au NW)	NA	223	100
	Bontapalle et al.	PEDOT : PSS	Au nanomembrane (Au NM, on S,D) /Carbon (Gate)	NA	56	30
	Li et al.	PEDOT : PSS	Au NM (S,D) /Carbon (Gate)	NA	13	45
	Chen et al.	DPP-g2T	Au	NA	15.83	140
	**This work**	**PEDOT : PSS-Ionic liquid**	**Au NM**	**0.3**	**1.25**	**30 (without PBS)**

Supplementary Material Reference:

1. Marchiori, B.; Delattre, R.; Hannah, S.; Blayac, S.; Ramuz, M. Laser-Patterned Metallic Interconnections for All Stretchable Organic Electrochemical Transistors. *Sci. Rep.* **2018**, *8*, 8477.
2. Lee, W.; Kobayashi, S.; Nagase, M.; Jimbo, Y.; Saito, I.; Inoue, Y.; Yambe, T.; Sekino, M.; Malliaras, G.G.; Yokota, T.; et al. Nonthrombogenic, Stretchable, Active Multielectrode Array for Electroanatomical Mapping. *Sci. Adv*. **2018**, 4, eaau2426.
3. Zhang, S.; Hubis, E.; Tomasello, G.; Soliveri, G.; Kumar, P.; Cicoira, F. Patterning of Stretchable Organic Electrochemical Transistors. *Chem. Mater*. **2017**, *29*, 3126–3132.
4. Dai, Y.; Dai, S.; Li, N.; Li, Y.; Moser, M.; Strzalka, J.; Prominski, A.; Liu, Y.; Zhang, Q.; Li, S.; et al. Stretchable Redox-Active Semiconducting Polymers for High-Performance Organic Electrochemical Transistors. *Adv. Mater*. **2022**, *34*, 2201178.
5. Bontapalle, S.; Na, M.; Park, H.; Sim, K. Fully Soft Organic Electrochemical Transistor Enabling Direct Skin-Mountable Electrophysiological Signal Amplification. *Chem. Commun*. **2022**, *58*, 1298–1301.
6. Li, Y.; Zhang, S.; Li, X.; Unnava, V.R.N.; Cicoira, F. Highly Stretchable PEDOT: PSS Organic Electrochemical Transistors Achieved via Polyethylene Glycol Addition. *Flex. Print. Electron.* **2019**, *4*, 44004.
7. Chen, J.; Huang, W.; Zheng, D.; Xie, Z.; Zhuang, X.; Zhao, D.; Chen, Y.; Su, N.; Chen, H.; Pankow, R.M.; et al. Highly Stretchable Organic Electrochemical Transistors with Strain-Resistant Performance. *Nat. Mater.* **2022**, *21*, 564–571.
